# Bridging gaps in transposable element research with single-molecule and single-cell technologies

**DOI:** 10.1186/s13100-018-0140-5

**Published:** 2018-12-06

**Authors:** Claudia Kutter, Patric Jern, Alexander Suh

**Affiliations:** 1 Department of Microbiology, Tumor and Cell Biology, Karolinska Institute, Science for Life Laboratory, SE-171 77 Stockholm, Sweden; 2 Science for Life Laboratory, Department of Medical Biochemistry and Microbiology, Uppsala University, SE-751 23 Uppsala, Sweden; 3 Department of Evolutionary Biology, Evolutionary Biology Centre, Science for Life Laboratory, Uppsala University, SE-752 36 Uppsala, Sweden

**Keywords:** Transposable elements, Repetitive sequence, Endogenous viruses, Satellites, Centromeres, Evolution, Long read-sequencing

## Abstract

More than half of the genomic landscape in humans and many other organisms is composed of repetitive DNA, which mostly derives from transposable elements (TEs) and viruses. Recent technological advances permit improved assessment of the repetitive content across genomes and newly developed molecular assays have revealed important roles of TEs and viruses in host genome evolution and organization. To update on our current understanding of TE biology and to promote new interdisciplinary strategies for the TE research community, leading experts gathered for the 2^nd^ Uppsala Transposon Symposium on October 4–5, 2018 in Uppsala, Sweden. Using cutting-edge single-molecule and single-cell approaches, research on TEs and other repeats has entered a new era in biological and biomedical research.

About 100 participants from all career stages attended the symposium (Fig. 1, https://transposonsymposium.wordpress.com/, #UppTransposon2018). The highly dynamic and diverse community of TE researchers was represented by gender-balanced contributions of the invited and selected abstract speakers.

**Fig. 1:**
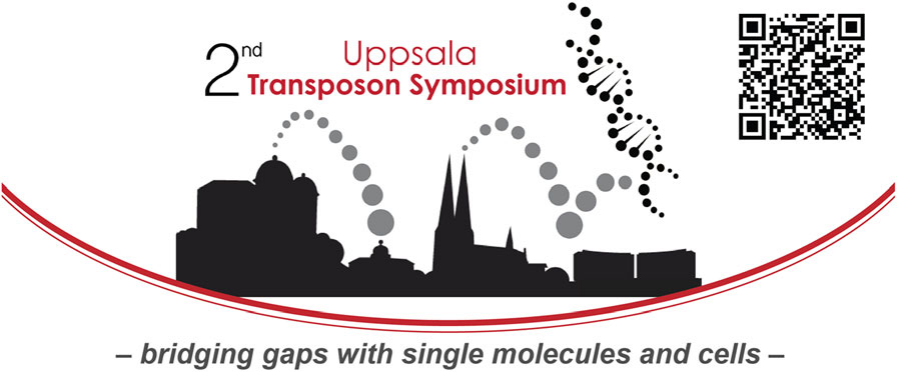
Symposium logo by Mercè Montoliu Nerin

## Revisiting genomic repeat annotations using single-molecule sequencing

Classical Sanger sequencing and more recently developed short-read sequencing have contributed substantially to genome research. However, accurate annotations are often hampered by high-copy repeat sequences such as transposable elements (TEs). Recent developments in long-read sequencing and other single-molecule technologies now allow better assessments of repetitive and complex genomic regions. Using a combination of available state-of-the-art technologies, several talks highlighted major advancements regarding genome assemblies, annotations and improvements of the genomic regulatory element catalogue. For instance, **Matthew Webster** (Uppsala University) and his group de novo assembled the honeybee *Apis mellifera* genome (bioRxiv; 10.1101/361469). To overcome allelic limitations presented by diploid organisms, the Webster team took advantage of drones, which are naturally haploid. The group first generated contigs based on PacBio sequencing, which were then merged with linked reads obtained from 10x Chromium. The Webster team finally built high-quality scaffolds by combining BioNano optical genome maps with Hi-C chromatin interaction maps and genetic linkage maps. Applying this comprehensive strategy, the group has now resolved complex structures across centromeres and telomeres and identified hundreds of AvaI and AluI repeats within these regions. This approach also allows the team to measure meiotic recombination rates. Similarly, using long read whole genome assemblies of the ascomycete fungus *Podospora anserina*, **Aaron Vogan** and Lorena Ament-Velasquez (postdoctoral researcher and PhD student, respectively, in Hanna Johannesson’s group, Uppsala University) have characterized the diversification of the meiotic drive gene family in fungi that are called spore killers (Spoks). Spok homologous genes reside in genomic stretches (blocks) of a highly complex structure that is up to 240 kb long. The Spok block is hypothesized to move around the genome through a DNA transposon-like mechanism. Remarkably, the Spok block might represent one of the largest selfish genetic elements.

**David Adelson** (University of Adelaide, @DavidAdelson3) and his group identify TE insertions in mammalian genomes by combining PacBio long read data with reads excluded from current assemblies. By using a sophisticated remapping approach (available on github.com/kortschak/loopy), the team provides updated annotations. The new annotations show that the number of somatic and germline TE insertions is underestimated. Although transposable elements make up a large proportion of most genomes, **Jullien Flynn** (PhD student in Andrew Clark’s group, Cornell University, @JullienFlynn) found that in some *Drosophila* species, simple satellite repeats dominate. Using PacBio and Illumina sequencing combined with cytogenetics, she showed that although there are biases against simple satellites in sequencing data, they make up almost half of the *Drosophila virilis* genome. The unprecedented power of long-read (“big whale”) over short-read (“small fishes”) sequencing was also featured in the keynote talk by **Karen Miga** (UC Santa Cruz, @khmiga). She has taken on the challenge to resolve the linear sequence of human centromeric regions. These regions are notoriously difficult to investigate since they are paved with arrays of repeated sequences. For the first time, the Miga group has provided a high-resolution study of chromosome-assigned satellite array repeats within the centromeric region. To investigate the nature of centromere sequence evolution, the group analyzed repeat composition and copy number variation between individuals. Utilizing PacBio long-read assemblies of 12 human genomes, the group has identified 36 common repeat array structures and 21 fast evolving satellite arrays. The latter might contribute to diversity within the human population. Similar to human, *Drosophila melanogaster* centromeres are also enriched in tandem satellite repeats. **Amanda Larracuente** (University of Rochester) and her collaborators in Barbara Mellone’s group (University of Connecticut) enrich for these regions using CENP-A chromatin immunoprecipitation followed by sequencing. They align the obtained reads to long-read whole genome assemblies and cross-check the results with fluorescence in situ hybridization techniques. Using this elegant strategy, they revealed that centromeres consist of TE-rich sequences (named after Italian islands) surrounded by large blocks of satellite arrays. Extending this work to other *Drosophila* species, the team found varying degrees of conservation, stressing the importance of repeat sequences as key driver of evolution.

Along these lines, **Megan Dennis** (UC Davis, @meganamsu) and her team use a combination of long-read sequencing techniques to investigate if genomic structural variation (deletion, insertions, duplications or inversions) contributes to genome evolution. She found that complex genomic rearrangements are likely mediated by great-ape-specific common “core” duplicated regions, including human-specific segmental duplications (> 98% sequence identity over 1 kb). As a consequence, new genes and regulatory elements are created and, surprisingly, often linked to human neurological disorders. To understand the contribution of repeat sequences to human malignancies and to discern cell type heterogeneity, **Rebecca Berrens** (postdoctoral researcher in John Marioni’s group, University of Cambridge, @Rberrens) pushed the boundaries of TE detection. She has optimized experimental and computational methods for Oxford Nanopore Technology (ONT) long-read sequencing to measure TE expression at a single-cell level with high fidelity and confidence. Using this sophisticated barcoding and error-correction strategy, she found that L1 elements are heterogeneously expressed across mouse embryonic stem cells. The development of a new strategy for linking short sequencing reads by **David Redin** (PhD student in Afshin Ahmadian’s group, Royal Institute of Technology, Stockholm, @Dreadin88) will also contribute to an improved resolution of genetic variation through sequencing. He developed a transposition-based assay to barcode millions of long DNA fragments to effectively reconstruct genomes with megabase-scale haplotypes and call structural variants (bioRxiv; 10.1101/356121). This truly remarkable technology not only reduces the cost for linked-read library preparation to about $19 per sample but also works astonishingly well for a wide range of sample types and input material, ranging from picograms to nanograms of DNA.

## “Selfish” repeat sequences across the tree of life

Given that repeat sequences are typically abundant in organismal genomes, researchers have been dissecting their contribution to genome evolution and to genetic diversity, which are often linked to biotic and abiotic environmental cues. For example, repetitive sequences can derive from viruses that insert their genetic material into the host genome. These “latent” sequences can contribute to evolutionary fitness. By using a BLAST algorithm-based comparative genomics approach, **Robert Gifford** (University of Glasgow, @Paleovirologist) inspects the evolution of viruses (in particular Hepadnaviruses) and repetitive sequences derived from these viruses. Infectious viruses that infiltrate the germline can be a source of new TEs. With the development of a number of tools (Database-integrated genome screening (DIGS) for endogenous viral elements (EVEs)), he showed that hepadnavirus-like retroelements (HEARTS) may have been horizontally transferred into wasps to form a symbiotic relationship.

In two fascinating talks, **Michelle Stitzer** (PhD student in Jeffrey Ross-Ibarra’s group, UC Davis, @mcstitzer) and **Mayela Soto** (PhD student in Magnus Nordborg’s group, GMI Vienna, @lmayela_soto) presented the contribution of TEs to genetic and phenotypic diversity in maize (*Zea mays*) and Arabidopsis (*Arabidopsis thaliana*) genomes, respectively. Using machine learning approaches and various other methods, both presenters determined the age and ancestry of TEs (https://github.com/mcstitzer/maize_v4_te_annotation). Their data suggest that a TE belonging to one family can jump into an already existing TE family. This symbiotic setting not only creates diverse TE families in plants but also provides new genomic features that are important for the survival in different ecosystems and geographic locations.

## Resolving transposon-mediated regulatory roles

Having established that TEs can arise in organismal genomes through either horizontal gene transfer or from viral germline infections followed by inheritance as endogenous viruses, the question remains how TE sequences can be repurposed for new functions in the host. In his keynote talk, **Cedric Feschotte** (Cornell University, @CedricFeschotte) reviewed the biological impact of TEs and focused on TE-derived protein-coding genes. He summarized a recently published study illustrating how a retrotransposon *Gag* gene was repurposed in the common ancestor of tetrapod vertebrates to function as a neuronal protein, called ARC, involved in memory storage. The ARC protein forms virus-like capsid structures, which package their own RNA and shuttle these transcripts from one neuron to another. In another, so far unpublished story of TE cooption, he described how a mariner transposase became fused to a Krüppel-associated box zinc-finger protein (KRAB-ZFP) to form a new gene specific to the lineage of vesper bats. This KRAB-mariner fusion protein has the ability to repress gene expression, and this activity may have been recruited to suppress expression of related transposons in the bat genome and possibly also the genes adjacent to them. These remarkable examples provide evidence that TE sequences can become incorporated into existing cellular and molecular programs to fuel the emergence of new biological processes.

A number of TEs are species-specifically expressed. **Johan Jakobsson** (Lund University, @JakobssonLab) and his team set out to investigate how L1 elements are controlled in human neural progenitor cells. He reasoned that DNA and histone methylation can keep evolutionarily young TEs under control. Indeed, the expression of L1 (and LTR12C) elements increases when DNA methyltransferase 1 (DNMT1) is deleted. In addition to DNA methylation, young TEs can be also silenced by TRIM28-mediated establishment of local heterochromatin (H3K9me3). This repressive local environment modulates protein-coding transcription, thereby altering regulatory networks essential for brain development and facilitates species-specific variation in brain gene expression programs. To investigate whether TEs are activated, for example, in response to stress conditions, **Simone Fouché** (PhD student in Bruce McDonald’s group, ETH Zurich, @SimoneFouche) studies TE expression in different isolates of the wheat pathogen *Zymoseptoria tritici*. She monitored TE gene expression at different time points and stress conditions induced by the host immune defense as well as upon nutrient starvation. Her results showed that inactive TEs become de-repressed, often within close proximity to protein-coding genes. Gypsy elements are activated by effects of the host immune defense whereas the expression of an uncharacterized TE peaks during nutrient starvation. Together, these findings suggest that TEs can shape the gene expression landscape. **Claudia Köhler** (Linnean Center for Plant Biology, Uppsala, @ClaudiaKohler11) investigates how TE activity can be repressed when neither heterochromatin nor DNA methylation control their expression. In collaboration with the teams of Rob Martienssen (Cold Spring Harbor Laboratory) and Keith Slotkin (University of Missouri), her team found that a special class of epigenetically-activated small interfering RNAs (easiRNAs) is generated by a novel pathway and protects the plant germline from TE invasion, similar to PIWI-interacting RNAs in mammalian systems. Interestingly, the Köhler team revealed that easiRNAs form a quantitative signal for chromosome number and that differences in easiRNA dosage contribute to the deregulation of paternally-expressed imprinted genes (PEGs). Disparities in chromosome number and thus easiRNA dosage impairs hybrid seed viability, thus connecting differences in TE number and composition with the rapid establishment of cross-species hybridization borders.

## Future perspective

Insightful comments regarding future TE research surfaced during the Q&A after the talks and in the concluding panel discussions of each conference day. Long-read and other single-molecule sequencing has certainly overcome a number of obstacles. Yet, many clusters of repetitive sequences are still unresolvable since individual reads cannot be linked correctly. Further technological advances, for example increased sequence read lengths extending from 1 Mb to a futuristic length of a whole chromosome, promise to overcome some of these issues. However, even the best genome assembly and alignment strategy still depends on robust annotation. Repbase is the most commonly used database for repeat annotations, but is far from complete. Utilizing additional specialized repeat identification and annotation tools as well as mining other database resources would improve the results. Efforts should be put into a unified classification as the current nomenclature often does not reflect phylogeny or is inconsistent between organisms. Furthermore, standardized analyses rather than irreproducible custom pipelines (despite code deposition) should be implemented. With the vast amounts of data generated each day, the TE research field is challenged by scaling up their analyses accordingly. Machine learning approaches or artificial intelligence may help to understand such complex data, yet models need to be trained entailing the danger of overfitting. Ultimately, the outcomes need to be evaluated independently or, in part, curated manually. Rising single-cell and single-molecule technologies (such as Hi-C) in combination with CRISPR screens might not only provide an alternative test system but could also overcome the choice of arbitrary cut-offs and contribute to more consistency in data analyses.

The biggest breakthroughs in TE research have been made through pure exploratory studies, curiosity, and serendipity. Nevertheless, funding agencies direct research activities towards immediately practical applications. Therefore, it is expected that future TE research will focus on addressing eminent societal challenges; including, explaining causes of and providing treatments for human diseases and aging-associated processes, maintenance of biodiversity, and sustainability in food security. Furthermore, engaging future generations in TE research is crucial. As most undergraduate studies skip over TE biology entirely, perhaps text book chapters can be improved and updated.

Understanding the mysterious world of TEs is complex and often common approaches are not applicable but it is exactly this challenge that gets researchers excited about studying TEs. The participants of the 2^nd^ Uppsala Transposon Symposium are certainly intrigued by investigating what they do not understand and are eager to hunt for more undiscovered factors and mechanisms. A stronger collaboration across disciplines, especially biology and biomedicine, will likely accelerate these efforts and developments. We thus consider this symposium an inspiration for TE research towards fostering interdisciplinary collaboration as well as knowledge exchange across career stages and scientific backgrounds.

## Abbreviations

DBD: DNA binding domain
DNMT: DNA methyltransferase
easiRNAs: Epigenetically-activated small interfering RNAs
ONT: Oxford Nanopore Technology
PacBio: Pacific Biosciences
PEGs: Paternally expressed imprinted genes
TE: Transposable element

